# Engagement Patterns of Dermatology TikTok Content by Creator Profession: Cross-Sectional Study

**DOI:** 10.2196/100102

**Published:** 2026-11-13

**Authors:** Grace Turchetta, Afrin Mirza, Matthew Keller

**Affiliations:** 1 Sidney Kimmel Medical College, Thomas Jefferson University, 1025 Walnut St, Philadelphia, PA, 19107, United States, 1 (215) 955-6983; 2 Department of Dermatology, Thomas Jefferson University Hospital, Philadelphia, PA, United States

**Keywords:** dermatology, social media, TikTok, health communication, patient education, online engagement

## Abstract

This cross-sectional analysis of 100 highly viewed dermatology-related TikTok videos found that influencers without medical training produced the majority of top-viewed content but did not differ significantly from dermatologists in engagement rate.

## Introduction

Social media is an important channel for sharing medical information, and dermatology-related content continues to expand online [1]. Dermatologists can use these platforms to educate large audiences [2], but individuals without formal medical training also produce dermatologic content of variable reliability [1-3].

TikTok, a short-form video platform, hosts a large volume of dermatology-related content. Research examining TikTok posts related to skin conditions found that most highly viewed videos were created by nonlicensed individuals [4]. Similarly, cross-sectional analyses of sun protection and skin-related content on TikTok found that dermatologists account for a minority of top-viewed videos [5,6]. The present study extends this work by evaluating engagement rates across creator professions using a hashtag-based sampling approach spanning multiple content categories, with the goal of better characterizing the dermatologic information landscape patients navigate when either seeking dermatologic guidance online or merely casually scrolling.

## Methods

A cross-sectional content analysis of dermatology-related TikTok videos was conducted on January 25, 2026. Five hashtags were selected from TikTok’s website based on their ranking as the most frequently used dermatology-related tags at the time of data collection: #dermatology, #skincare, #sensitiveskin, #antiagingskincare, and #acne. This preliminary cross-section was not intended to be exhaustive. For each hashtag, the 20 most-viewed videos were identified, yielding 100 videos for analysis. No duplicate videos were identified across hashtags. Data collection was performed using a newly created TikTok account with no prior viewing history to minimize algorithmic personalization bias.

For each video, total views, likes, comments, and shares were recorded. Median values for each metric were calculated across videos within each creator category. The engagement rate was calculated as the sum of likes, comments, and shares divided by total views and expressed as a percentage. Creators were categorized based on publicly available profile information as (1) dermatologists, (2) nonphysician skin professionals, (3) influencers without formal medical training, or (4) other. Because viewers cannot readily verify credentials, creators were classified by how they presented themselves. We attempted to confirm dermatologists’ board certification through the American Board of Dermatology, CertificationMatters.org, and a Google search of each creator’s name; certification was confirmed for 7 of 10. Groups were compared using Kruskal-Wallis tests, with Dunn post hoc comparisons (Holm adjusted); *P*<.05 was considered significant.

## Results

Among the 100 videos analyzed, 63 (63%) were created by influencers without medical training, 25 (25%) by nonphysician skin professionals, 10 (10%) by dermatologists, and 2 (2%) by creators in the other category (1 nondermatologist physician and 1 pre–physician assistant student). These 2 videos are presented in Table 1 but excluded from cross-group comparisons given the small sample size.

**Table 1.** Number of videos and median views, likes, comments, shares, and engagement rate by creator type.

	Videos, n/100 (%)	Likes, median (IQR)	Views, median (IQR)	Comments, median (IQR)	Shares, median (IQR)	Engagement rate, %
Influencer	63 (63)	186,400 (52,000-579,900)	2,900,000 (1,150,000-8,900,000)	930 (305-2384)	23,000 (5952-64,500)	8.5263
Nonphysician skin professional	25 (25)	28,800 (8,040-143,200)	775,600 (254,600-4,300,000)	675 (201-1738)	7399 (1227- 20,300)	6.4914
Nondermatologist physician^a^	1 (1)	44,100	492,800	326	2329	—^b^
Physician assistant student^a^	1 (1)	97,900	1,600,000	79	3517	—^b^
Dermatologist	10 (10)	56,450 (6522.25-263,750)	2,200,000 (401,075-6,725,000)	746 (170-2,498.75)	18,750 (5,269.75-120,125)	5.3991

a Individual values shown.

b Not applicable given n=1.

Influencers had the highest median views (2,900,000), likes (186,400), comments (930), and shares (23,000), followed by dermatologists (2,200,000 views; 56,450 likes) and nonphysician skin professionals (Table 1).

Engagement rates were highest among influencers (8.53%), followed by nonphysician skin professionals (6.49%). Dermatologist-produced videos had an engagement rate of 5.40%. Groups differed in views (*P*=.03), likes (*P*=.001), and shares (*P*=.045), but not comments (*P*=.50) or engagement rate (*P*=.19). Differences were between influencers and nonphysician skin professionals; dermatologists did not differ significantly from either group. Excluding the 3 dermatologists whose board certification could not be confirmed did not alter the direction or statistical significance of these findings.

A subgroup analysis compared creator distribution across medical hashtags (#dermatology, #sensitiveskin, and #acne; n=60) and beauty-oriented hashtags (#skincare and #antiagingskincare; n=40). Dermatologists created 9/60 (15.0%) of videos under medical hashtags vs 1/40 (2.5%) under beauty-oriented hashtags (influencers: 39/60, 65.0% vs 24/40, 60.0%; nonphysician skin professionals: 10/60, 16.7% vs 15/40, 37.5%; Table 2). Dermatologists were most represented under #dermatology (8/20, 40%), and absent under #acne, where influencers created 19/20 (95%) and nonphysician skin professionals 1/20 (5%).

**Table 2.** Number of videos^a^ in each category by hashtag and creator type.

All videos and hashtag	Dermatologist, n (%)	Influencer, n (%)	Nonphysician skin professional, n (%)	Other^b^, n (%)	Total, n
All videos	10 (10)	63 (63)	25 (25)	2 (2)	100
#dermatology	8 (40)	2 (10)	8 (40)	2 (10)	20
#sensitiveskin	1 (5)	18 (90)	1 (5)	0 (0)	20
#acne	0 (0)	19 (95)	1 (5)	0 (0)	20
#skincare	0 (0)	18 (90)	2 (10)	0 (0)	20
#antiagingskincare	1 (5)	6 (30)	13 (65)	0 (0)	20

a Percentages were calculated using a denominator of 100 for “All videos” or 20 for each hashtag.

b Nondermatology physician (n=1) and physician assistant student (n=1).

## Discussion

Most highly viewed dermatology-related TikTok videos were created by influencers without formal medical training, while relatively few were produced by dermatologists. These findings align with prior research demonstrating that nonlicensed individuals generate much of the highly viewed dermatologic content on social media [4]. Although dermatologists produced fewer videos, their content still achieved high median view counts, indicating physician-generated content can reach large audiences when present. Moreover, dermatologist videos had numerically lower engagement rates than influencer videos, but this difference was not statistically significant.

The finding that dermatologists account for only 10% of top-viewed videos warrants contextual interpretation. Dermatologists represent fewer than 4 per 100,000 people in the United States [7]. From this perspective, dermatologists’ share of top-viewed videos may be notable for a numerically small specialty, although the number of dermatologists actively posting on TikTok is unknown. Moreover, several of the hashtags included, such as #skincare and #antiagingskincare, span both medical and consumer beauty domains, where influencer participation is expected. Dermatologists were best represented under #dermatology (40%) but absent under the top 20 most-viewed #acne videos, a condition squarely within dermatologic expertise, whereas influencers accounted for 95% of content. Because acne is one of the most common conditions for which patients seek dermatologic care, the absence of physician-generated content may have meaningful implications for patient education and treatment decisions.

Dermatology is the most visible medical specialty on social media and only a small percentage of dermatologists actively create content [8]. Thus, it faces a unique challenge and opportunity to influence patient education before they walk into the clinic.

The study has several limitations. Board certification could not be confirmed for 3 of 10 dermatologist creators, and credentials of nonphysician skin professionals were largely self-reported. Findings were unchanged when the 3 unconfirmed creators were excluded, and viewers are unlikely to verify credentials themselves. The nonphysician skin professional group was heterogeneous, and licensure could not be independently verified for all creators, so it was not analyzed by subcategory. The 5 hashtags were selected based on reported frequency of use from TikTok’s website; pilot data guiding this selection would have enhanced methodological reproducibility and the hashtags may not be representative of the full breadth of dermatologic content on the platform. Some hashtags are more associated with consumer beauty interests rather than medical dermatology. Although a newly created account minimized algorithmic bias, recommendation systems may still influence results, and the dynamic nature of engagement metrics limits reproducibility. Creator responsiveness to comments, which is associated with platform visibility and may be harder for clinicians to sustain, was not captured. Video content and viewer comments were not analyzed. Finally, engagement metrics reflect popularity rather than educational value or information accuracy. Follow-up studies are intended to assess content accuracy and comment themes.

## References

[1] DeBord LC, Patel V, Braun TL, Dao H (2019). Social media in dermatology: clinical relevance, academic value, and trends across platforms. J Dermatolog Treat.

[2] Sierro TJ, Young PM, Kassabian SK, Wu KK, Armstrong AW (2020). Dermatologists in social media: a study on top influencers, posts, and user engagement. J Am Acad Dermatol.

[3] Ranpariya V, Chu B, Fathy R, Lipoff JB (2020). Dermatology without dermatologists? Analyzing Instagram influencers with dermatology-related hashtags. J Am Acad Dermatol.

[4] Devjani S, Ezemma O, Fruechte S (2023). “Skinfluencers” versus dermatologists as creators of the top dermatology-related videos on TikTok. J Clin Aesthet Dermatol.

[5] Lin RR, Pulumati A, Woolery-Lloyd H (2024). DermTok: who’s talking sun? A cross-sectional analysis of sun protection content on TikTok. J Drugs Dermatol.

[6] Campbell J, Williams K, Woolery-Lloyd H (2023). DermTok: How TikTok is changing the landscape of dermatology patient education. J Drugs Dermatol.

[7] Glazer AM, Rigel DS (2017). Analysis of trends in geographic distribution of US dermatology workforce density. JAMA Dermatol.

[8] Correia E, Mandel J, Cullison SRJ (2025). Analyzing social media trends in cosmeceuticals: insights from Google Trends and TikTok Analytics. J Cosmet Dermatol.

